# Immunotherapy and Overall Survival Among Patients With Advanced Non–Small Cell Lung Cancer and Obesity

**DOI:** 10.1001/jamanetworkopen.2024.25363

**Published:** 2024-08-02

**Authors:** Yasutaka Ihara, Kenji Sawa, Takumi Imai, Tsubasa Bito, Yuki Shimomura, Ryota Kawai, Ayumi Shintani

**Affiliations:** 1 Department of Medical Statistics, Graduate School of Medicine, Osaka Metropolitan University, Osaka, Japan; 2 Department of Clinical Oncology, Graduate School of Medicine, Osaka Metropolitan University, Osaka, Japan

## Abstract

**IMPORTANCE:**

The association between obesity and response to cancer treatment and survival remains unclear, with conflicting findings from various studies. The optimal choice between conventional chemotherapy and immunotherapy for first-line treatment remains uncertain in patients with obesity who potentially have an inadequate therapeutic response to immunotherapy.

**OBJECTIVE:**

To investigate whether body mass index (BMI) modifies the association of immunotherapy or conventional therapy with overall survival in patients with advanced non–small cell lung cancer (aNSCLC).

**DESIGN, SETTING, and PARTICIPANTS:**

A retrospective cohort study, using administrative claims data obtained from advanced treatment centers in Japan, was conducted between December 1, 2015, and January 31, 2023. Participants included individuals aged 18 years or older with aNSCLC who received immunotherapy, using immune checkpoint inhibitor (ICI) treatment or conventional chemotherapy.

**EXPOSURE:**

Immune checkpoint inhibitor therapy as first-line chemotherapy was compared with conventional chemotherapy, identified through patient medical records.

**MAIN OUTCOMES AND MEASURES:**

The main outcome was overall survival. Survival analysis covered a 3-year follow-up period after the first-line chemotherapy.

**RESULTS:**

A total of 31 257 patients with aNSCLC were identified. Of these, 12 816 patients received ICI therapy (mean [SD] age, 70.2 [9.1] years; 10 287 [80.3%] men) and 18 441 patients received conventional chemotherapy (mean [SD] age, 70.2 [8.9] years; 14 139 [76.7%] men). Among patients with BMI less than 28, ICI therapy was associated with a significantly lower hazard of mortality (eg, BMI 24: hazard ratio [HR], 0.81; 95% CI, 0.75-0.87) compared with those who underwent conventional chemotherapy. However, no such association was observed among patients with BMI 28 or greater (eg, BMI 28: HR, 0.90; 95% CI, 0.81-1.00).

**CONCLUSIONS AND RELEVANCE:**

The findings of this retrospective cohort study suggest that BMI modifies the association of ICI therapy compared with conventional chemotherapy with overall survival in patients with aNSCLC. A lack of association between ICI therapy and improved survival in patients with aNSCLC and overweight or obesity compared with conventional chemotherapy was observed. This suggests that ICI therapy may not be the optimal first-line therapy for patients with overweight or obesity and the use of conventional chemotherapy should also be considered in such patients.

## Key Points

**Question:**

For patients with advanced non–small cell lung cancer (aNSCLC) and obesity who potentially have an inadequate therapeutic response to anti–programmed cell death 1 therapy, is conventional chemotherapy or immune checkpoint inhibitor the optimal choice as first-line therapy?

**Findings:**

In this cohort study, 31 257 patients with aNSCLC were identified. Immune checkpoint inhibitor was not associated with improved survival compared with conventional chemotherapy in patients with aNSCLC and overweight or obesity.

**Meaning:**

The findings of this study suggest that immune checkpoint inhibitor therapy may not be the optimal first-line therapy in patients with overweight or obesity and aNSCLC; hence, the use of conventional chemotherapy should also be considered in such patients.

## Teaser

This cohort study compares the use of immune checkpoint inhibitor therapy with conventional chemotherapy in patients with non–small cell lung cancer (NSCLC) and overweight or obesity.

## Introduction

The number of patients with overweight or obesity is increasing worldwide.^1^ Obesity has been reported as a risk factor for solid tumors and lifestyle-related diseases, such as hypertension and diabetes.^2,3,4,5,6,7^ Patients with cancer and obesity are generally recognized to have a less-favorable prognosis than their counterparts who have a normal weight. Therefore, both the American Cancer Society^8^ and the European Society for Medical Oncology^9^ advocate implementing weight management strategies among cancer survivors. However, several observational studies have reported that patients with cancer and a higher body mass index (BMI) experience improved survival compared with patients with normal weight and cancer receiving chemotherapy.^10,11,12,13,14,15,16,17,18^ This discrepancy is known as the obesity paradox^10^ and exists not only in patients with cancer receiving conventional chemotherapy,^11,12,13^ but also in those undergoing treatment with immune checkpoint inhibitors (ICIs).^14,15,16,17,18^ For example, compared with patients with normal weight who had non–small cell lung cancer (NSCLC) and received ICI therapy, the overall survival hazard ratio (HR) was 0.64 (95% CI, 0.51-0.81) in patients with overweight and 0.81 (95% CI, 0.68-0.95) in those with obesity, with higher BMI associated with greater overall survival.^16^ The obesity paradox in patients with cancer can be explained by both clinical and methodologic factors.^10^ Clinically, less-aggressive tumor biologic factors in patients with obesity may play a role. For example, obesity is associated with less-aggressive molecular mutations in kidney carcinoma.^19^ Additionally, tumors in patients with obesity might respond better to therapy due to differences in pharmacokinetics and treatment allocation.^20^ Methodologically, confounding factors, such as smoking and physical activity, influence both obesity and mortality but are not fully adjusted for, skewing results.^21^ Detection bias occurs when incidental cancers are diagnosed during screenings for other obesity-related conditions, such as diabetes, leading to overestimated cancer survival rates.^22^ These cancers are generally low-stage cancers with a good prognosis and may be the cause of the obesity paradox in patients with obesity.

Preclinical studies have reported that obesity hampers the response to anti–programmed cell death 1 (PD-1) therapy.^23,24^ For instance, a preclinical study involving mice with intrakidney tumors receiving anti-PD-1–based immunotherapy reported a lower frequency of treatment responders in obese mice compared with lean mice.^23^ This study suggested that host obesity, rather than factors such as a high-fat diet, was responsible for the reduced efficacy of immunotherapy. The counts of CD4^+^ and CD8^+^ T cells in the mesenteric lymph nodes of obese mice were observed to be reduced compared with those in control mice. This finding suggests that obesity may diminish the effectiveness of ICI therapy, which primarily activates and targets CD8^+^ T cells.^24^ Conversely, it is unlikely that obesity would reduce the therapeutic efficacy in patients receiving conventional chemotherapy, such as cytotoxic anticancer agents. This is because these agents directly attack cancer cells and inhibit their division and proliferation. However, studies investigating the association between BMI and overall survival in patients with cancer and obesity who received ICI therapy vs conventional chemotherapy are lacking. Hence, the optimal choice between conventional chemotherapy and immunotherapy for first-line therapy remains uncertain in patients with obesity who potentially have an inadequate therapeutic response to anti-PD–1 therapy. Therefore, we hypothesized that conventional chemotherapy may be associated with greater therapeutic response than immunotherapy in patients with obesity and cancer.

In this study, we aimed to investigate whether BMI modifies the association of immunotherapy as a first-line therapy compared with conventional chemotherapy on overall survival in patients with advanced NSCLC (aNSCLC). Then, we explored the possibility of inadequate therapeutic responses to ICI therapy compared with conventional chemotherapy in patients with obesity and aNSCLC. To this end, we analyzed a large sample of patients with lung cancer in Japan using preexisting administrative claims data. There was underrepresentation of patients with NSCLC in the authorization clinical trials of ICI therapy compared with a clinical practice setting. This is another knowledge gap and reason to conduct retrospective cohort studies such as the present one to identify various effect modifiers and other important attributes of therapy in clinical practice settings.^25^

## Methods

### Data Source

In this retrospective cohort study, the data used were extracted from an administrative claims database (maintained by Medical Data Vision) for patients diagnosed with lung cancer (*International Statistical Classification of Diseases and Related Health Problems, 10th Revision* [*ICD-10*], code C34) between April 1, 2008, and January 31, 2023. The database combines data from more than 400 acute care hospitals, representing approximately 23% of all insurance claims in Japan. It registers information for approximately 38 million patients.^26^ The dataset used in this study included anonymized electronic health insurance claims and diagnostic procedure combinations, linking routinely collected information for each patient. Details covered include age, sex, height, weight, diagnosis based on *ICD-10* codes, medical procedures, prescription medications, and survival status, spanning both outpatient and inpatient medical care. The database has been used in other studies on lung cancer.^27,28^ The study followed the Strengthening the Reporting of Observational Studies in Epidemiology (STROBE) reporting guideline. The Japanese Ethical Guidelines for Medical and Biological Research Involving Human Subjects are not applied to clinical studies that use data anonymized before the start of the study.^29^ The data used in this study were anonymized by the database before the start of the study. Therefore, informed consent was waived.

### Study Population

The study included patients aged 18 years or older who received chemotherapy for new aNSCLC between December 1, 2015, and January 23, 2023. To identify patients who received first-line therapy after lung cancer diagnosis, we included those with a minimum 1-month interval from database entry to the date of first lung cancer diagnosis. In our study, the initiation of chemotherapy after the diagnosis of lung cancer was recorded as the index date (eMethods 1 in Supplement 1). To thoroughly assess the association between BMI and overall survival for both conventional chemotherapy and immunotherapy, patients receiving therapeutic drugs for driver mutations (eTable 1 in Supplement 1) on the index date were excluded. To identify patients with aNSCLC, those who received chemoradiotherapy or adjuvant chemotherapy were excluded.^30^ Furthermore, patients whose BMI could not be calculated due to missing height or weight data were excluded. The detailed exclusion criteria are reported in eMethods 2 in Supplement 1.

### Identification of BMI

Body mass index was calculated by dividing participants’ weight in kilograms by their height in meters squared. We used data on height and weight, which were measured within 1 month before the index date and recorded in a discharge summary. World Health Organization classes were used to categorize BMI: underweight, less than 18.5; normal weight, 18.5 to 24.9; overweight, 25.0 to 29.9; and obesity, 30.0 or greater.^31^

### Identification of Death

Survival status and date of death were obtained from the discharge summaries. For patients with confirmed deaths on the database, data were extracted up until their date of death, whereas those with unconfirmed deaths were censored at the last recorded date of the medical procedure. Survival status was collected for up to 3 years after the index date.

### Demographic and Clinical Information

Baseline covariates included clinical characteristics, concomitant medications, and underlying diseases, such as lifestyle-related diseases. eTable 2 in Supplement 1 summarizes the list of codes used to identify each disease or medical procedure. The process of identifying demographic and clinical characteristics is outlined in eMethods 3 in Supplement 1.

### Statistical Analysis

The baseline characteristics of patients who received conventional chemotherapy or ICI therapy were summarized with frequencies and proportions used for categorical variables, mean (SD) for normally distributed continuous variables, and median (IQR) for nonnormally distributed continuous variables. Body mass index distribution in the study population was visualized using histograms.

To examine whether BMI modifies the association between ICI therapy as a first-line therapy compared with conventional chemotherapy and overall survival in patients with aNSCLC, we used multivariable Cox proportional hazards regression models, including the type of therapy, BMI, and their cross-product term. Patients were classified by the type of first-line therapy they received for the entire follow-up period (eMethods 4 in Supplement 1). The continuous BMI value was included in the model using a restricted cubic spline function with 4 knots, and the following variables were adjusted: age, sex, histologic characteristics of NSCLC (squamous or nonsquamous), Charlson comorbidity index score,^32^ Barthel index score,^33^ smoking status, duration from December 1, 2015, to the index date, hypertension, dyslipidemia, and immunosuppressant therapy. The estimated log of hazard function for mortality was plotted on a logarithmic scale against continuous BMI values, and the possible association of type of therapy and mortality was assessed by computing specific BMI. Details of subgroup, sensitivity, and additional analyses conducted to examine the robustness of the main results are described in eMethods 4 in Supplement 1.

To address missing values of the Barthel index score and smoking status, both of which served as covariates in a series of regression analyses, multiple imputations were conducted, using predictive mean matching to generate the imputed dataset.^34^ Statistical significance was defined as 2-sided *P* < .05. All analyses were performed using R, version 4.2.2 (R Foundation for Statistical Computing) software.^35^

## Results

### Summary of the Study Population

**Figure 1.**
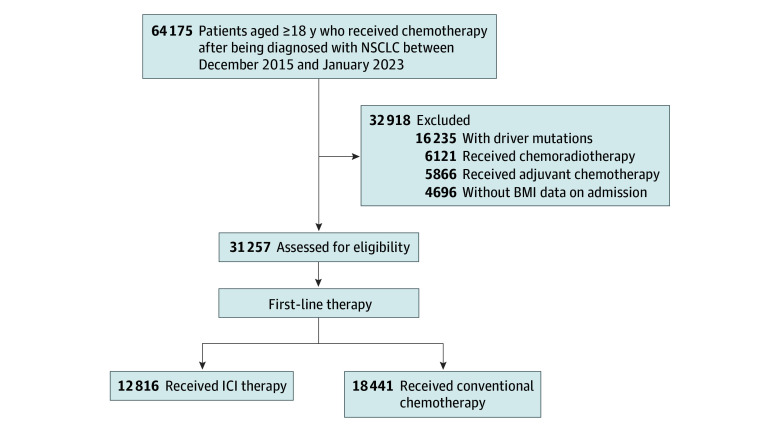
Flowchart of the Study Population BMI indicates body mass index; ICI, immune checkpoint inhibitor; and NSCLC, non–small cell lung cancer.

Figure 1 shows a flowchart of the patients in the study. We identified 64 175 adults (age, ≥18 years) with NSCLC between December 1, 2015, and January 31, 2023. Of 31 257 patients who met the eligibility criteria, 12 816 patients (mean [SD] age, 70.2 [9.1] years; mean [SD] BMI, 21.9 [3.5]; 10 287 [80.3%] men; 2529 [19.7%] women) received ICI therapy, and 18 441 patients (mean [SD] age, 70.2 [8.9] years; mean [SD] BMI, 22.1 [3.5]; 14 139 [76.7%] men; 4302 [23.3%] women) received conventional chemotherapy as first-line therapy. Among 12 816 patients who received ICI therapy, 77.3% (n = 9905) received pembrolizumab, 12.7% (n = 1630) received nivolumab (including ipilimumab), and the remaining 10.0% (n = 1281) received atezolizumab. eFigure 1 in Supplement 1 shows BMI distribution for the entire population and subgroups based on therapy types.

### Patient Characteristics

The Table presents the patients’ characteristics in this study categorized by weight class. Obesity was more common in younger patients than in the other ages. Moreover, the proportions of patients with underlying diabetes and dyslipidemia increased with increasing BMI. There were no significant differences in age or BMI between the groups receiving different first-line therapies (eTable 3 in Supplement 1). Patient characteristics are reported according to age category (eTable 4 in Supplement 1), sex (eTable 5 in Supplement 1), and details of therapy type (eTable 6 in Supplement 1). Patient characteristics with missing BMI appeared similar between groups receiving different first-line therapies (eTable 7 in Supplement 1).

**Table.** Characteristics of Patients With NSCLC Categorized According to Weight Class

Patient characteristic	No. (%)							
	Underweight^a^		Normal weight^a^		Overweight^a^		Obese^a^	
	ICI therapy (n = 2018)	Conventional chemotherapy (n = 2641)	ICI therapy (n = 8562)	Conventional chemotherapy (n = 12 409)	ICI therapy (n = 1977)	Conventional chemotherapy (n = 3011)	ICI therapy (n = 259)	Conventional chemotherapy (n = 380)
Age, mean (SD), y	70.2 (8.7)	69.8 (8.9)	70.3 (9.1)	70.5 (8.8)	70.2 (9.5)	69.9 (9.1)	67.3 (10.5)	66.6 (10.6)
BMI, mean (SD)	17.0 (1.2)	17.4 (1.2)	21.7 (1.8)	21.8 (1.8)	26.7 (1.3)	26.7 (1.3)	32.3 (2.4)	32.6 (2.9)
Sex								
Male	1489 (73.8)	1820 (68.9)	7008 (81.9)	9722 (78.3)	1606 (81.2)	2365 (78.5)	184 (71.0)	232 (61.1)
Female	529 (26.2)	821 (31.1)	1554 (18.1)	2687 (21.7)	371 (18.8)	646 (21.5)	75 (29.0)	148 (38.9)
Smoking status (current/past)^b^	1629 (84.2)	1981 (78.0)	6775 (81.9)	9287 (77.8)	1528 (79.4)	2259 (77.9)	185 (74.6)	266 (71.5)
Barthel index score (perfect score)^b^	1608 (84.2)	2152 (86.3)	7318 (89.5)	10 881 (90.9)	1742 (90.7)	2657 (91.5)	225 (90.0)	337 (90.8)
CCI score								
0-2	675 (33.4)	1074 (40.7)	2894 (33.8)	5307 (42.8)	710 (35.9)	1303 (43.3)	89 (34.4)	175 (46.1)
≥3	1343 (66.6)	1567 (59.3)	5668 (66.2)	7102 (57.2)	1267 (64.1)	1708 (56.7)	170 (65.6)	205 (53.9)
Histologic characteristics of NSCLC								
Squamous	395 (19.6)	444 (16.8)	1537 (18.0)	2058 (16.6)	344 (17.4)	504 (16.7)	41 (15.8)	56 (14.7)
Nonsquamous	1623 (80.4)	2197 (83.2)	7025 (82.0)	10 351 (83.4)	1633 (82.6)	2507 (83.3)	218 (84.2)	324 (85.3)
Underlying conditions								
Hypertension	698 (34.6)	802 (30.4)	3851 (45.0)	4932 (39.7)	1097 (55.5)	1505 (50.0)	163 (62.9)	218 (57.4)
Diabetes	327 (16.2)	418 (15.8)	1788 (20.9)	2671 (21.5)	509 (25.7)	854 (28.4)	85 (32.8)	131 (34.5)
Kidney failure	76 (3.8)	78 (3.0)	393 (4.6)	437 (3.5)	109 (5.5)	106 (3.5)	13 (5.0)	10 (2.6)
Liver failure	318 (15.8)	395 (15.0)	1434 (16.7)	1756 (14.2)	390 (19.7)	476 (15.8)	56 (21.6)	79 (20.8)
Cardiovascular disease	636 (31.5)	654 (24.8)	2843 (33.2)	3575 (28.8)	702 (35.5)	973 (32.3)	91 (35.1)	154 (40.5)
Dyslipidemia	301 (14.9)	357 (13.5)	2109 (24.6)	2817 (22.7)	672 (34.0)	874 (29.0)	94 (36.3)	125 (32.9)
Medication								
Immunosuppressants	22 (1.1)	79 (3.0)	71 (0.8)	349 (2.8)	16 (0.8)	74 (2.5)	2 (0.8)	10 (2.6)
Calendar dates of therapies								
December 1, 2015-December 31, 2019	681 (33.7)	1727 (65.4)	2897 (33.8)	8071 (65.0)	631 (31.9)	1906 (63.3)	83 (32.0)	227 (59.7)
January 1, 2020- January 31, 2023	1337 (66.3)	914 (34.6)	5665 (66.2)	4338 (35.0)	1346 (68.1)	1105 (36.7)	176 (68.0)	153 (40.3)

Abbreviations: BMI, body mass index (calculated as weight in kilograms divided by height in meters squared); CCI, Charlson comorbidity index; ICI, immune checkpoint inhibitor; NSCLC, non–small cell lung cancer.

^a^ World Health Organization BMI classes: underweight, less than 18.5; normal weight, 18.5 to 24.9; overweight, 25.0 to 29.9; and obese, 30.0 or greater.

^b^ Data on smoking status were missing for 3.6% of the total population and on Barthel index for 4.0% of the total population.

### Results of Main Analyses

**Figure 2.**
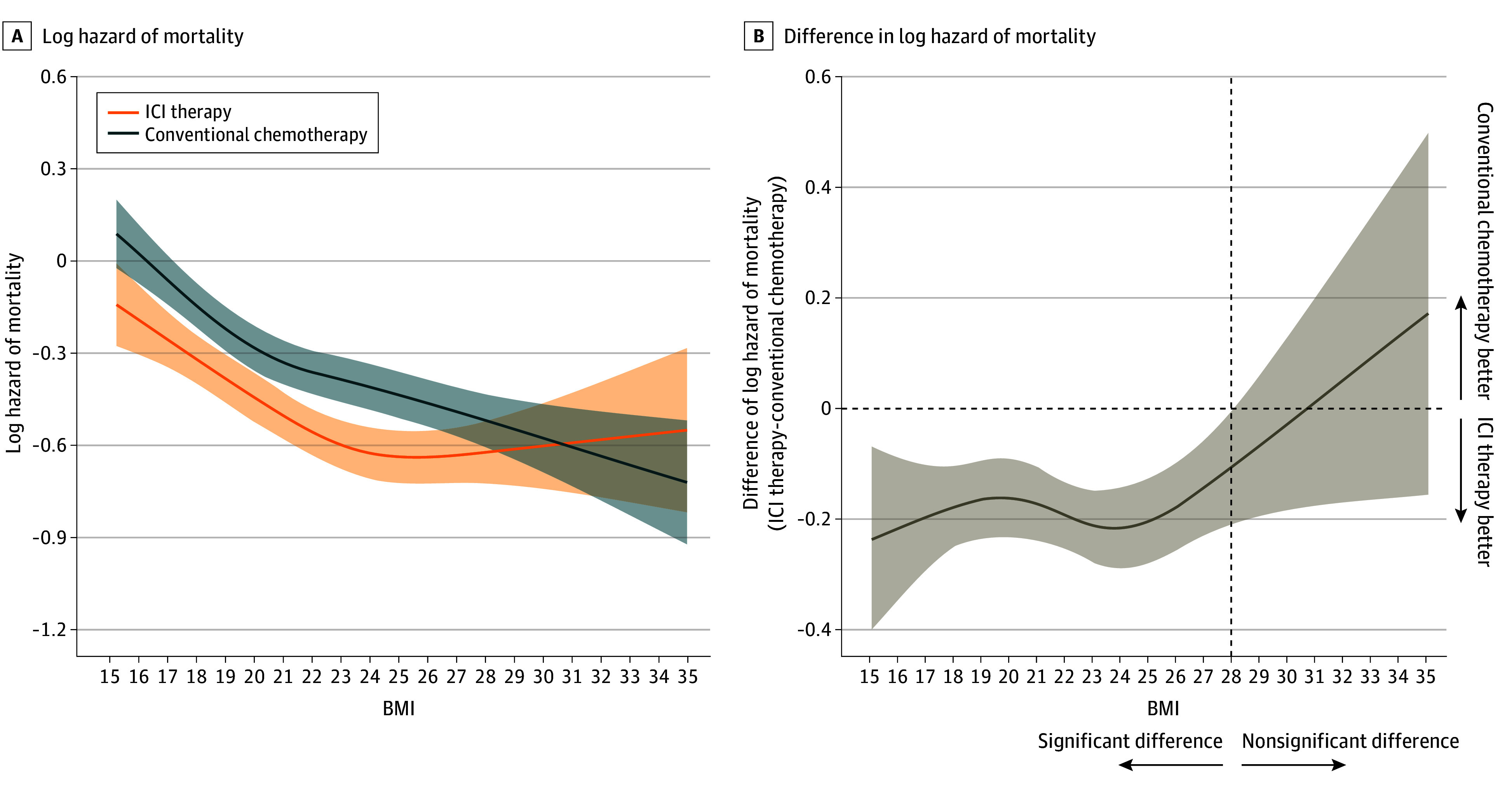
Hazard Functions of Mortality With Immune Checkpoint Inhibitor (ICI) Therapy vs Conventional Chemotherapy and Difference of Hazard of Mortality Plotted Against Body Mass Index (BMI) A, Hazard functions of mortality for patients who received ICI therapy (n = 12 816) or conventional chemotherapy (n = 18 441). *P* < .001 for BMI (calculated as weight in kilograms divided by height in meters squared) association with both ICI therapy and conventional chemotherapy. B, Difference of hazards of mortality plotted against BMI. Multivariable Cox proportional hazards regression, including the type of therapy, BMI, and their cross-product term. The following variables were adjusted for other covariates: age, sex, histologic characteristics of non–small cell lung cancer (squamous or nonsquamous), Charlson comorbidity index score, Barthel index score, smoking status, duration from December 1, 2015, to the index date, hypertension, dyslipidemia, and immunosuppressant therapy.

The proportion of patients who died within 3 years of first-line therapy was 28.0% (3586 of 12 816) in patients receiving ICI therapy and 35.9% (6627 of 18 441) in those receiving conventional chemotherapy. Figure 2A shows a log of mortality hazard as a function of BMI, stratified by therapy type. For both patients receiving conventional chemotherapy or ICI therapy, there was an association between the type of therapy and BMI on a log of mortality hazard of BMI (*P* < .001 for both analyses).

A nonlinear association of BMI on a log of mortality hazard was observed in patients who received conventional chemotherapy (*P*<.001) and ICI therapy (*P* = .001). In both therapy types, an association was observed between a higher BMI and a lower mortality hazard compared with a lower BMI. Among patients who received ICI therapy, the hazard of mortality was associated with a steady decrease as BMI increased from 15 to 24; however, at BMI greater than 24, the risk was associated with an increase, indicating a U-shaped association between BMI and mortality. Figure 2B shows BMI-specific differences in a log of mortality hazard in patients receiving ICI therapy compared with those receiving conventional chemotherapy as a function of BMI. Body mass index–specific HR of mortality comparing 2 therapy types was statistically significant for BMI of less than 28 (eg, BMI 24: HR, 0.81; 95% CI, 0.75-0.87) and HR for BMI of 28 or greater was not statistically significant (eg, BMI 28: HR, 0.90; 95% CI, 0.81-1.00) (Figure 2B).

### Results of Subgroups, Sensitivity, and Additional Analyses

**Figure 3.**
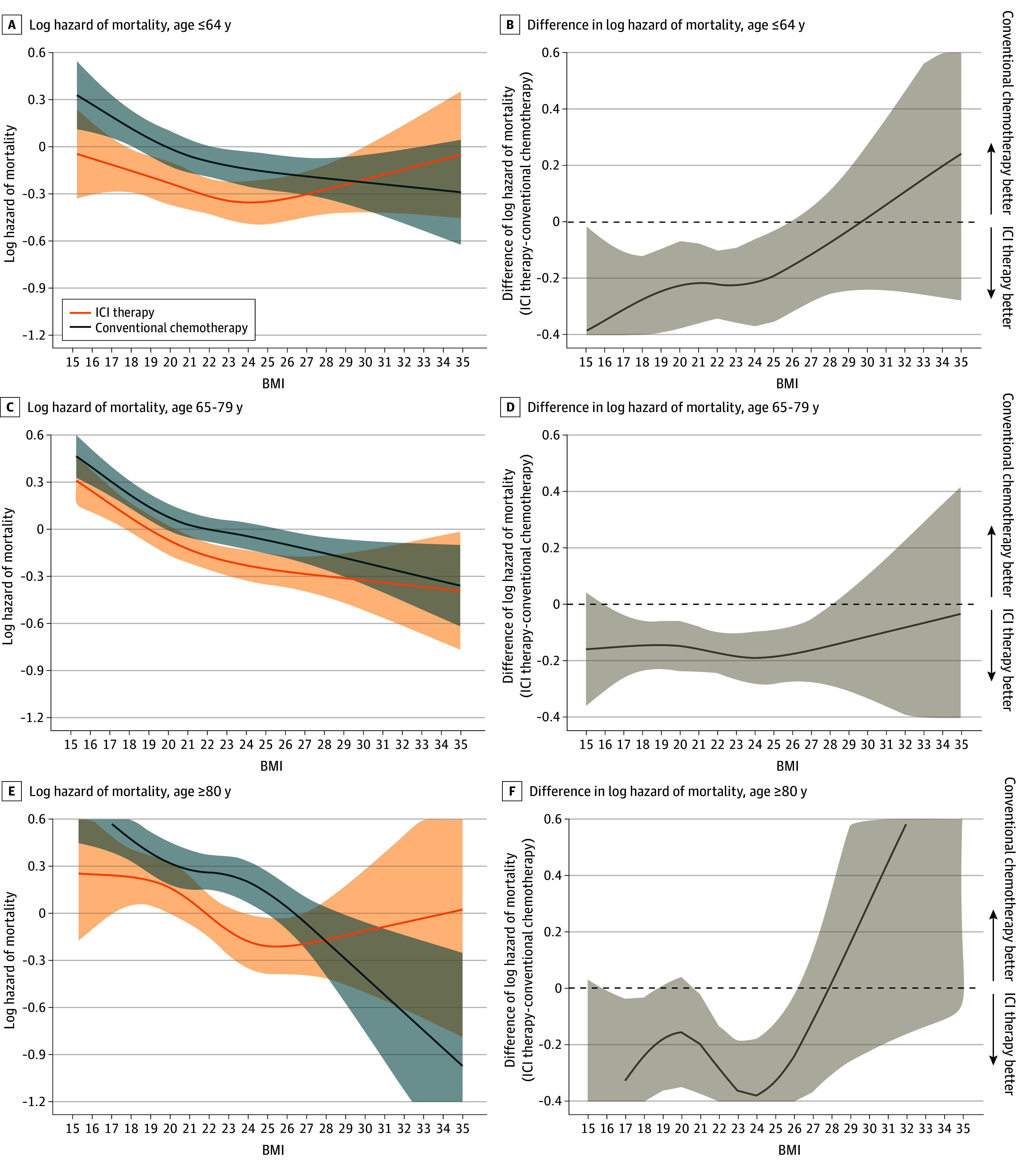
Hazard Functions of Mortality With Immune Checkpoint Inhibitor (ICI) Therapy vs Conventional Chemotherapy and Difference of Hazard of Mortality Plotted Against Body Mass Index (BMI) Stratified by Age Category Log hazard mortality (A) and difference in log hazard mortality (B) in patients aged 64 years or younger receiving ICI therapy (n = 2835) or conventional chemotherapy (n = 3938). Log hazard mortality (C) and difference in log hazard mortality (D) in patients aged 65 to 79 years receiving ICI therapy (n = 8290) or conventional chemotherapy (n = 12 190). Log hazard mortality (E) and difference in log hazard mortality (F) in patients aged 80 years or older receiving ICI therapy (n = 1691) or conventional chemotherapy (n = 2313). Multivariable Cox proportional hazards regression, including the type of therapy, BMI (calculated as weight in kilograms divided by height in meters squared), age category, and their cross-product term. The following variables were adjusted for other covariates: sex, histologic characteristics of non–small cell lung cancer (squamous or nonsquamous), Charlson comorbidity index score, Barthel index score, smoking status, duration from December 1, 2015, to the index date, hypertension, dyslipidemia, and immunosuppressant therapy.

**Figure 4.**
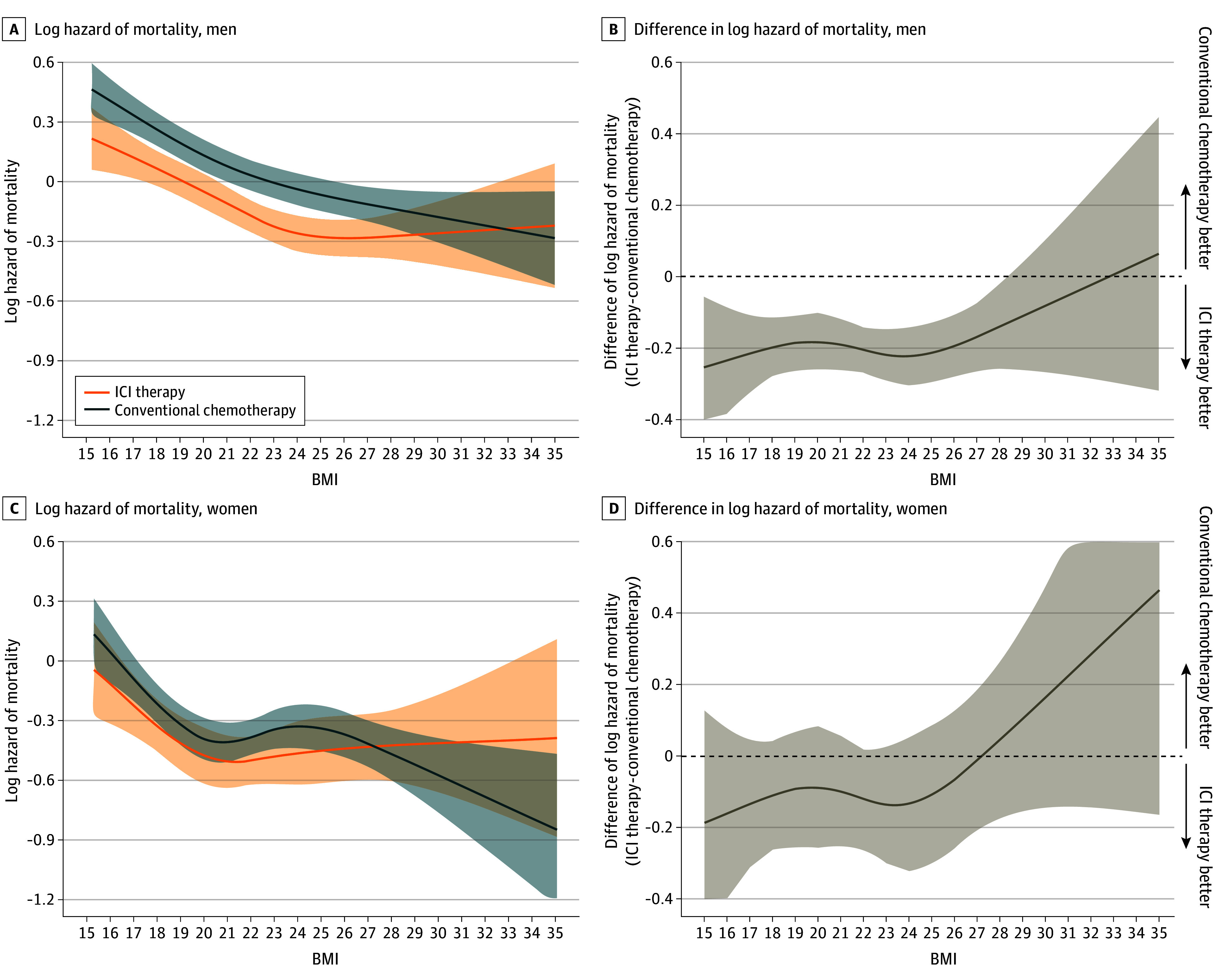
Hazard Functions of Mortality for Patients Who Received Immune Checkpoint Inhibitor (ICI) Therapy or Conventional Chemotherapy and Difference of Hazard of Mortality Plotted Against Body Mass Index (BMI) Stratified by Sex Log hazard mortality (A) and difference in log hazard mortality (B) in men receiving ICI therapy (n = 10 287) or conventional chemotherapy (n = 14 139). Log hazard mortality (C) and difference in log hazard mortality (D) in women receiving ICI therapy (n = 2529) or conventional chemotherapy (n = 4302). Multivariable Cox proportional hazards regression, including the type of therapy, BMI (calculated as weight in kilograms divided by height in meters squared), sex, and their cross-product term. The following variables were adjusted for other covariates: age, histologic characteristics of non–small cell lung cancer (squamous or nonsquamous), Charlson comorbidity index score, Barthel index score, smoking status, duration from December 1, 2015, to the index date, hypertension, dyslipidemia, and immunosuppressant therapy.

Figure 3 and Figure 4 show a log of mortality hazard as a function of BMI, stratified by therapeutic type within the male and female subpopulations. A consistent association with a lower hazard of mortality for patients with a higher BMI was observed in both therapy types for all age and sex subgroups (Figure 3A, C,E; Figure 4A, C). The association of a lower hazard of mortality for patients receiving ICI therapy compared with those receiving conventional chemotherapy was observed to disappear around overweight or obesity in all age subgroups (Figure 3B, D, F). These associations were also observed in men, but were less clear in women (Figure 4B, D).

The results of the sensitivity and additional analyses are shown in eFigure 2 and eFigure 3 in Supplement 1. The results of all sensitivity analyses were consistent with those in Figure 2B of the main analysis.

## Discussion

This study found that, among patients with aNSCLC who received ICI therapy or conventional chemotherapy, overweight or obesity was associated with a lower risk of mortality than a lower BMI. Additionally, this study suggests that BMI modifies the association of ICI therapy as a first-line therapy compared with conventional chemotherapy on overall survival in patients with aNSCLC. A BMI less than 28 in patients who received ICI therapy was associated with a significantly lower hazard of mortality compared with those receiving conventional chemotherapy. However, this association was not observed in patients with BMI greater than or equal to 28.

Our findings further suggest that, among patients with aNSCLC who received ICI therapy or conventional chemotherapy, those with overweight or obesity had a lower risk of mortality than those with a lower BMI, as shown in Figure 2A. These results support the presence of the obesity paradox in patients with aNSCLC who underwent either therapy. In previous observational studies that evaluated nonlinearity with BMI as a continuous variable, patients with unresectable or metastatic melanoma and aNSCLC who received ICI therapy showed a U-shaped association between BMI and mortality, with the lowest risk observed at a BMI of 25 to 34.^14,36^ Consistent with previous studies,^14,36^ our study showed a U-shaped association between BMI and mortality in patients with aNSCLC who received ICI therapy. However, in our study, the lowest hazard of mortality was observed at a BMI of 24. Beyond this threshold, the hazard of mortality gradually increased at a slower rate compared with previous studies.^14,36^ This difference may be due to substantial variations in BMI distribution between ethnicities, with a lower BMI in most patients in Japan compared with the US.^31^ Additionally, the present study may have been limited in its ability to accurately characterize the association between mortality and obesity in patients, as the proportion of individuals with a BMI greater than 30 was relatively low compared with previous studies.^14,36^

The results of this study support the preclinical findings^23,24,37,38,39^ suggesting that patients with obesity may exhibit inadequate therapeutic responses to anti-PD-1 therapy, as shown in a U-shaped association between BMI and mortality in patients with aNSCLC who received ICI therapy (Figure 2B). The expression of PD-1 on CD8^+^ T cells, which influences the efficacy of immunotherapy, has been reported to be modulated by various factors, such as age, female hormones, and gut microbiota.^40,41,42^ For instance, G-protein–coupled estrogen receptor agonists have been shown to synergize with anti-PD1 immunotherapy by increasing immunogenicity.^43^ In this study,^43^ the mean age of women who received ICI therapy was 70.1 years, suggesting that most women were postmenopausal. This implies that immunotherapy was limited owing to the decrease in estrogen production in postmenopausal individuals.^44^ As expected, the association of a lower hazard of mortality in women with aNSCLC who received ICI therapy was not significant for any BMI range. Thus, the association of ICI therapy in patients with obesity may be affected differently by variations in patient characteristics. The reason why patients with aNSCLC who received ICI therapy or conventional chemotherapy exhibited an association between a decreased hazard of mortality and increasing BMI is unclear. This association was observed with conventional chemotherapy irrespective of age and sex, suggesting potential stability compared with ICI therapy. Further studies are needed to determine which patient characteristics would increase efficacy in patients with obesity.

### Limitations

This study has some limitations. First, the data used were not specifically collected for research purposes but were derived from existing clinical practice information, not allowing for a randomized clinical trial. Second, obesity was evaluated based on BMI, a metric widely used to assess body fat percentage but incapable of distinguishing between muscle and adipose tissue.^11^ Patients with cancer who are overweight and have higher muscle mass reportedly experience a major survival advantage, emphasizing the need for further research to elucidate the effect of body composition on cancer mortality.^45,46^ However, muscle storage is likely to be equally biased in patients with aNSCLC who received ICI therapy and conventional chemotherapy. Third, data on deaths other than in-hospital deaths were not available in the database. We do not believe that missing posthospital death information occurs differently between patients receiving conventional chemotherapy and immunotherapy; therefore, misclassification of the outcome may be limited. Fourth, PD-L1 status could not be determined from this database. Hence, there is a possibility that patients with low or negative PD-L1 expression were more likely to receive conventional chemotherapy. Fifth, we investigated the association between BMI and overall survival in patients receiving conventional chemotherapy or ICI therapy, handling BMI as a continuous variable instead of a categorical one as in previous studies. We did not adjust for multiple comparisons in our statistical analysis, which raises the possibility of inflation of type I error. Sixth, the results of this study are based on data from the Japanese population. Therefore, the generalizability of these findings to other populations may be limited.

## Conclusions

This cohort study showed a lack of association between immunotherapy and improved survival in patients with aNSCLC and overweight or obesity compared with conventional chemotherapy. This finding supports the possibility that patients with obesity may have an inadequate response to immunotherapy. This suggests that ICI therapy may not necessarily be the optimal first-line therapy for patients with overweight or obesity, and the use of conventional chemotherapy should also be considered.

## Notes

Supplement 1. **eMethods 1.** Definitions of Patients With NSCLC Who Received First-Line Therapy **eMethods 2.** Patient Exclusion Criteria **eMethods 3.** Definitions of Patient Demographic and Clinical Characteristics **eMethods 4.** Detailed Information on Each Analysis **eTable 1.** *ATC* Codes of Anticancer Drugs for Driver Mutation Identification **eTable 2.** List of Codes for *ICD-10* and Medical Procedures **eTable 3.** Characteristics of Patients With NSCLC Categorized According to Therapy Types **eTable 4.** Characteristics of Patients With NSCLC Categorized According to Age **eTable 5.** Characteristics of Patients With NSCLC Categorized According to Sex **eTable 6.** Characteristics of Patients With NSCLC Categorized According to Details on Therapy Types **eTable 7.** Characteristics of NSCLC Patients With and Without BMI Values **eFigure 1.** Distribution of BMI **eFigure 2.** Hazard Functions of Mortality for Patients Who Received ICI Therapy or Conventional Chemotherapy and Difference of Hazard of Mortality Plotted Against BMI in Sensitivity Analyses **eFigure 3.** Hazard of Mortality as a Function of BMI Stratified Based on Details on Therapy Types

Supplement 2.
Data Sharing Statement
