# Supplementary material for: Immunotherapy and Overall Survival Among Patients With Advanced Non–Small Cell Lung Cancer and Obesity
Source: JAMA Netw Open. 2024 Aug 2;7(8):e2425363. doi: 10.1001/jamanetworkopen.2024.25363 (PMC11297387; doi:10.1001/jamanetworkopen.2024.25363)
Supplement: Supplement 1. — eMethods 1. Definitions of Patients With NSCLC Who Received First-Line Therapy eMethods 2. Patient Exclusion Criteria eMethods 3. Definitions of Patient Demographic and Clinical Characteristics eMethods 4. Detailed Information on Each Analysis eTable 1. ATC Codes of Anticancer Drugs for Driver Mutation Identification eTable 2. List of Codes for ICD-10 and Medical Procedures eTable 3. Characteristics of Patients With NSCLC Categorized According to Therapy Types eTable 4. Characteristics of Patients With NSCLC Categorized According to Age eTable 5. Characteristics of Patients With NSCLC Categorized According to Sex eTable 6. Characteristics of Patients With NSCLC Categorized According to Details on Therapy Types eTable 7. Characteristics of NSCLC Patients With and Without BMI Values eFigure 1. Distribution of BMI eFigure 2. Hazard Functions of Mortality for Patients Who Received ICI Therapy or Conventional Chemotherapy and Difference of Hazard of Mortality Plotted Against BMI in Sensitivity Analyses eFigure 3. Hazard of Mortality as a Function of BMI Stratified Based on Details on Therapy Types [file jamanetwopen-e2425363-s001.pdf]

Supplementary Online Content

Ihara Y, Sawa K, Imai T, et al. Immunotherapy and overall survival among patients with advanced non–small cell lung cancer and obesity. *JAMA Netw Open*. 2024;7(8):e2425363. doi:10.1001/jamanetworkopen.2024.25363

eMethods 1. Definitions of Patients With NSCLC Who Received First-Line Therapy

eMethods 2. Patient Exclusion Criteria

eMethods 3. Definitions of Patient Demographic and Clinical Characteristics

eMethods 4. Detailed Information on Each Analysis

eTable 1. *ATC* Codes of Anticancer Drugs for Driver Mutation Identification

eTable 2. List of Codes for *ICD-10* and Medical Procedures

eTable 3. Characteristics of Patients With NSCLC Categorized According to Therapy Types

eTable 4. Characteristics of Patients With NSCLC Categorized According to Age

eTable 5. Characteristics of Patients With NSCLC Categorized According to Sex

eTable 6. Characteristics of Patients With NSCLC Categorized According to Details on Therapy Types

eTable 7. Characteristics of NSCLC Patients With and Without BMI Values

eFigure 1. Distribution of BMI

eFigure 2. Hazard Functions of Mortality for Patients Who Received ICI Therapy or Conventional Chemotherapy and Difference of Hazard of Mortality Plotted Against BMI in Sensitivity Analyses

eFigure 3. Hazard of Mortality as a Function of BMI Stratified Based on Details on Therapy Types

This Supplementary material has been provided by the authors to give readers additional information about their work.

© 2024 Ihara Y et al. *JAMA Network Open.*

eMethods 1. Definitions of patients with NSCLC who received first-line therapy

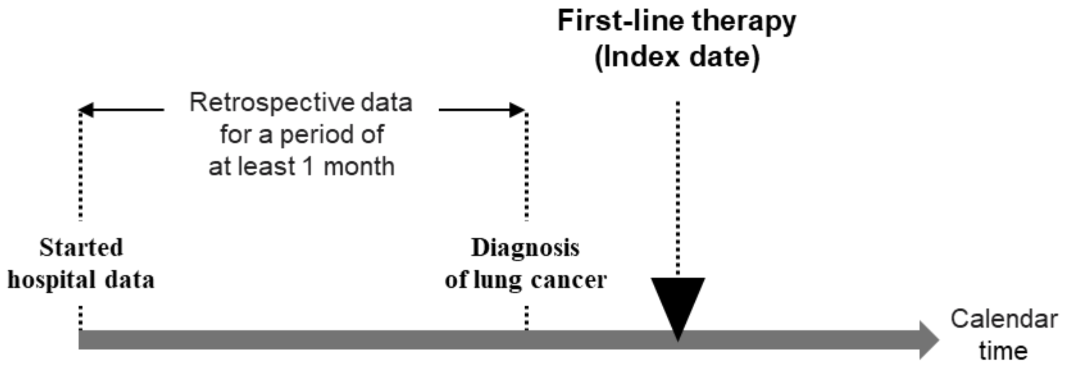

To identify patients who received first-line therapy after lung cancer diagnosis, we included those with a minimum 1-month interval from database entry to the date of first lung cancer diagnosis. In our study, the initiation of chemotherapy after the diagnosis of lung cancer was recorded as the index date.

Abbreviations: NSCLC, non-small cell lung cancer

© 2024 Ihara Y et al. *JAMA Network Open.*

eMethods 2. Patient exclusion criteria

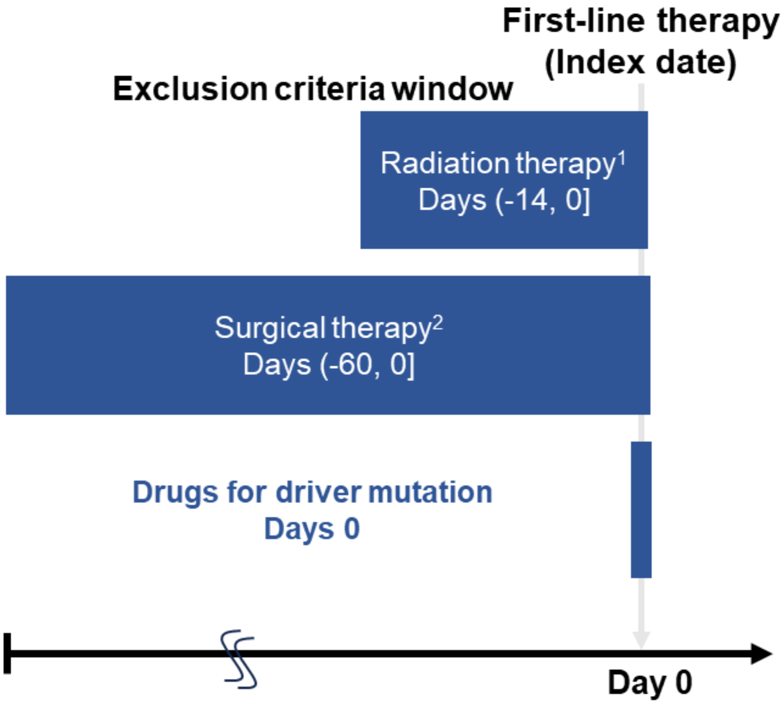

[1] List of codes for radiation therapy

	Medical procedures codes
Radiation therapy	180019410, 180019710, 180020710, 180020810, 180020910, 180021010, 180021110, 180021210, 180021310, 180021410, 180021510, 180021610, 180021710, 180021810, 180021910, 180022010,180026750, 180031910

[2] List of codes for surgical therapy

	Medical procedures codes
Surgical therapy	150357810, 150357910, 150358010, 150358110, 150358210, 150358310, 150358410, 150358510, 150358610, 150358710, 150358810, 150374510, 150386610, 150406110, 150414410

Patients were excluded from the study if they met any of the following criteria: (1) received chemoradiotherapy within 14 days before the index date; (2) received adjuvant chemotherapy within 60 days before the index date; (3) received a therapeutic agent targeting a driver mutation on the index date.

© 2024 Ihara Y et al. *JAMA Network Open.*

eMethods 3. Definitions of patient demographic and clinical characteristics

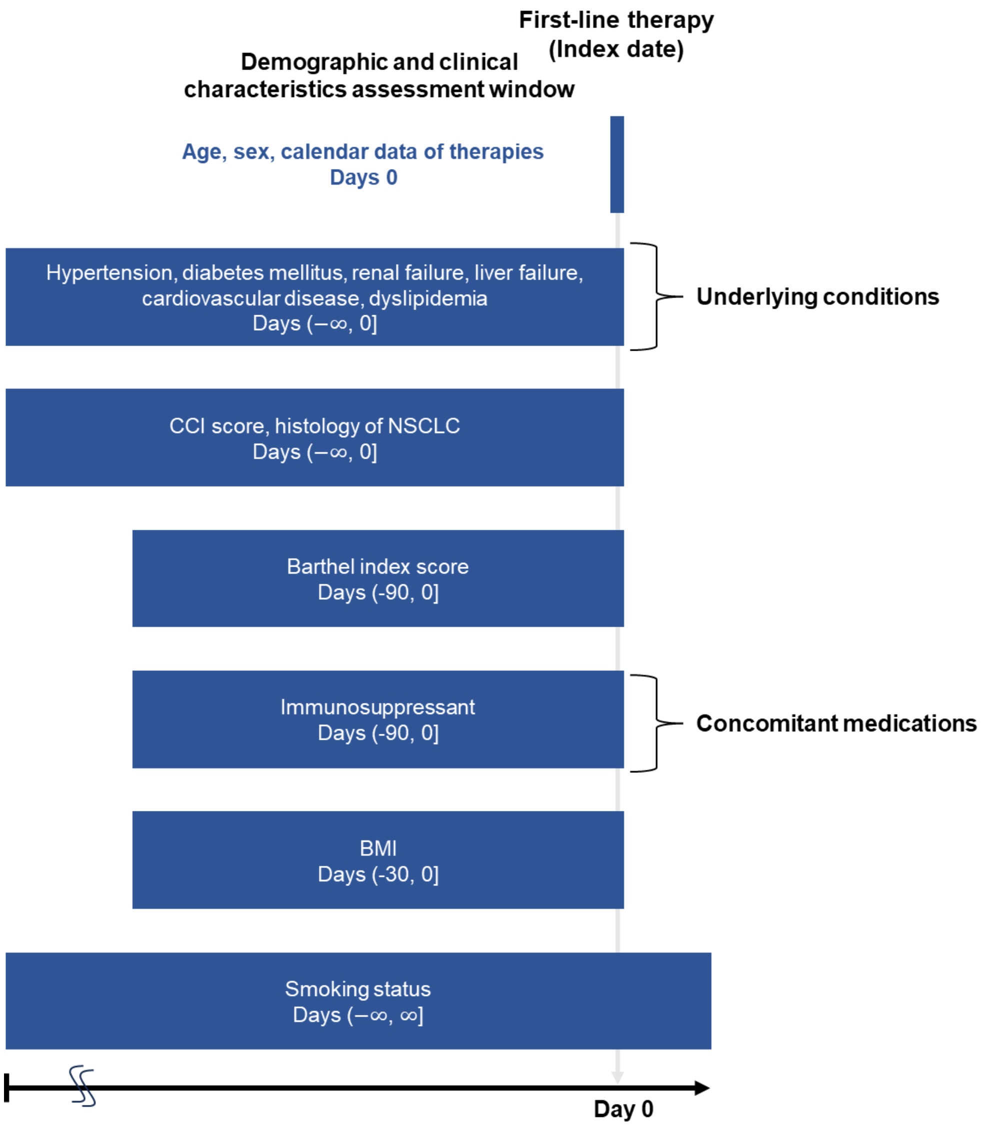

Based on the index date, age, sex, and calendar dates of therapies were collected. BMI was collected within 1 month prior to the index date. Barthel index score and medications (immunosuppressant) were collected within 3 months prior to the index date. Additionally, data available before the index date including underlying conditions (hypertension, diabetes mellitus, renal failure, liver failure, cardiovascular disease, dyslipidemia), Charlson comorbidity index (CCI) score, and histology of NSCLC (squamous/non-squamous) were collected. Smoking status was collected using data before and also after the index date because the information regarding past smoking history may appear after the index date.

Abbreviations: NSCLC, non-small cell lung cancer; CCI, Charlson comorbidity index; BMI, body mass index

© 2024 Ihara Y et al. *JAMA Network Open.*

eMethods 4. Detailed information on each analysis

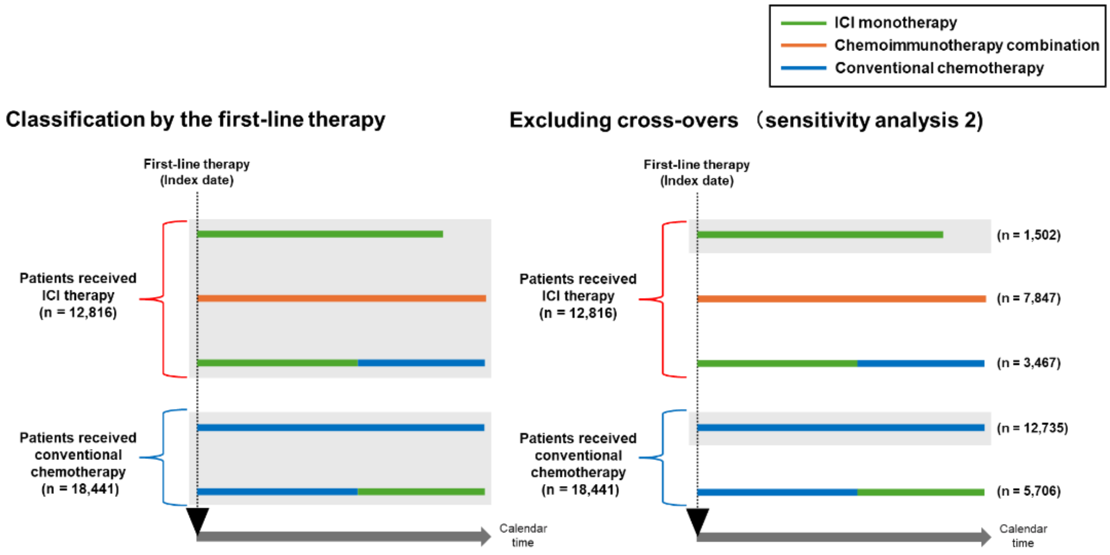

The analysis population for each therapy is grey-filled.

Main analysis of Classification by the first-line therapy

Method which is labled as "Classification by the first-line therapy" analyzed patients' data for the entire follow-up period according to their first choice of ICI therapy or conventional chemotherapy after the index date ignoring mid-course switching their therapy.

Subgroups analyses by age category and sex

To account for the potential influence of age and sex on the relationship between BMI and mortality, the main analyses were repeated within subgroups of patients categorized by age (≤ 64 years, 65–79 years, and ≥ 80 years) and sex (male and female).

Sensitivity analysis 1 on length of period before lung cancer

Two sensitivity analyses were conducted to examine the robustness of the main results by changing the following definitions. We included those with a minimum 3-month interval from database entry to the date of first lung cancer diagnosis (sensitivity analysis 1).

Sensitivity analysis 2 with censoring at time of regimen cross-over

As a sensitivity analysis 2, we also conducted a method of "Excluding cross-overs" in which we excluded patients who switched over their regimens during a follow-up period. This sensitivity analysis included patients who received ICI monotherapy and conventional chemotherapy. If a patient receiving ICI monotherapy, started conventional chemotherapy, he or she was excluded from the analysis. Similarly, if a

© 2024 Ihara Y et al. *JAMA Network Open.*

patient receiving conventional chemotherapy, started ICI therapy, he or she was excluded from the analysis. Patients receiving ICI monotherapy (n = 1,502) and conventional chemotherapy (n = 12,735) in the "Excluding cross-over" shown below were included (sensitivity analysis 2).

Sensitivity analysis 3 in missing value completion of BMI

4,696 patients were excluded from the analysis due to missing BMI information. Missing data on BMI were caused by participating hospitals' decision on providing or not providing BMI information to MDV. Patients' baseline characteristics among patients with missing BMI were compared between ICI and conventional chemotherapy groups (Appendix eTable 6). The distributions of all baseline characteristics appeared to be similar between the two cohorts. We also have added a sensitivity analysis using multiple imputation methods. Multiple imputation with predictive mean matching was used to address missing values of BMI in addition to Barthel index score and smoking status, and analysis was performed on the imputed dataset (sensitivity analysis 3).

Additional analysis

As an additional analysis, ICI therapy was further divided into chemoimmunotherapy combination (combination therapy of ICI and conventional chemotherapy) and ICI monotherapy (including in nivolumab + ipilimumab). The estimated hazard function of mortality was plotted on a log scale against continuous BMI values.

Abbreviations: ICI, Immune checkpoint inhibitor; BMI, body mass index

© 2024 Ihara Y et al. *JAMA Network Open.*

eTable 1. *ATC* codes of anticancer drugs for driver mutation identification

KEGG DGROUP	ATC codes	Anticancer drugs names
EGFR inhibitors	L01EB01	Gefitinib
	L01EB02	Erlotinib
	L01EB03	Afatinib
	L01EB04	Osimertinib
	L01EB07	Dacomitinib
BRAF inhibitors	L01EC02	Dabrafenib
	L01EE01	Trametinib
ALK inhibitors	L01ED01	Crizotinib
	L01ED02	Ceritinib
	L01ED03	Alectinib
	L01ED04	Brigatinib
	L01ED05	Lorlatinib
MET inhibitors	L01EX21	Tepotinib
	L01EX17	Capmatinib
RET inhibitor	L01EX22	Selpercatinib
NTRK inhibitors	L01EX14	Entrectinib
	L01EX12	Larotrectinib

Abbreviations: ATC, Anatomical therapeutic chemical; EGFR, epidermal growth factor receptor; BRAF, B-Raf serine-threonine kinase; ALK, anaplastic lymphoma kinase; MET, mesenchymal epithelial transition; RET, rearranged during transfection; NTRK, neurotrophic tyrosine receptor kinase

© 2024 Ihara Y et al. *JAMA Network Open.*

eTable 2. List of codes for *ICD-10* and medical procedures

Underlying conditions	ICD-10 codes
Hypertension	I10, I11, I12, I13, I14, I15
Diabetes mellitus	E10, E11, E12, E13, E14
Renal failure	I12.0, I13.1, N03.2–N03.7, N05.2–N05.7, N18, N19, N25.0, Z49.0–Z49.2, Z94.0, Z99.2
Liver failure	B18.x, K70.0–K70.3, K70.9, K71.3–K71.5, K71.7, K73, K74, K76.0, K76.2–K76.4, K76.8, K76.9, Z94.4, I85.0, I85.9, I86.4, I98.2, K70.4, K71.1, K72.1, K72.9, K76.5, K76.6, K76.7
Cardiovascular disease	I01-I02.0, I05-I13, I20-I25, I27, I30-I51, I60-I69
Dyslipidemia	E785
**Medication**	**Medical procedure codes**
Immunosuppressant	620008997, 620009119, 621133002, 622023001, 622746800, 666210001, 610409338, 620008070, 620008106, 620008107, 620008509, 620008510, 620008511, 620008512, 620008513, 620008514, 620009440, 620009441, 620506801, 621867201, 621884302, 621884303, 621890601, 621965201, 621969902, 622011801, 622026201, 622053401, 622065002, 622102901, 622102902, 622102903, 622109401, 622646301, 614210169, 620005941, 622181601, 640453101, 644210037, 614210098, 620007515, 622221301, 644210048, 644210049, 620006808, 622093601, 622509701, 622509801, 622608801, 622608901, 622609001, 629911301, 629911401, 629913601, 629913701, 640462006, 622378801, 622577001, 622590801, 622596901, 622651501, 620002479, 620007231, 621934101, 622003901, 622242701, 622624701, 622635001, 622635101, 622635201, 629906301, 622676001, 622676101, 622676201, 622676301, 629900301, 629900501, 622070002, 622675801, 622224501, 622654701, 622508101, 622508201, 622046501, 622554701, 622123701, 622639401, 622403701, 622518901, 622608101, 629911101, 620006184, 620006804, 620006805, 620008445, 620008829, 622510201, 613990075, 613990088, 620004279, 620006560, 629916101, 622533801, 622533901, 622534001, 622793801, 622793901, 621984301, 620004854, 620004855, 620004856, 620008850, 610443018, 610443019, 610443020, 610443021, 613990074, 613990085, 613990086, 620002641,

© 2024 Ihara Y et al. *JAMA Network Open.*

Medication	Procedure codes
Immunosuppressant	620002728, 620002729, 620009313, 620009314, 620882601, 620894001, 621326201, 621483603, 621483703, 621637802, 621674701, 621674801, 621677601, 621677701, 621685602, 621732201, 621738001, 621743306, 621743406, 622043804, 622056201, 622056301, 622056401, 622741800, 622741900, 622742000, 622363701, 610409342, 610443059, 610451009, 610451010, 613990096, 620008437, 620008438, 620008439, 622047401, 622232201, 622232301, 622270501, 622270601, 622280901, 622281001, 622281101, 622281201, 622281301, 622370001, 622370101, 622384301, 622384303, 622384401, 622384403, 622384501, 622384503, 622384601, 622384603, 622384703, 622437901, 622438001, 622438101, 622438601, 622438701, 622580901, 622590901, 643990141, 622242601, 622573701, 622573801, 622440601, 622587101, 622587201, 622587301, 622587401, 622678101, 622678201, 610432045, 622272501, 622283901, 622440801, 613990069, 613990070, 621993201, 621993301, 622008902, 622009001, 622009002, 622518701, 622518801, 611140861, 611140862, 620003877, 620003878, 620003879, 620003880, 622002901, 622265701, 622491601, 622183501, 622184501, 620006160, 620006895, 620007135, 620942001, 610453008, 610453065, 610463004, 620004897, 621442802, 621442905, 621443001, 621443002, 622039002, 622103601, 622103602, 622161402, 622173801, 622173901, 622746900, 622747000, 622833801, 622596601, 622596701, 622662001, 622662101, 622242201, 622242301, 622830601, 622830701, 610422247, 610433106, 610433156, 620000180, 620000181, 620006092, 620008052, 620942203, 620942401, 620943101, 622108401, 622108402, 622743000, 622743100, 620005116, 620005117, 610432016, 620002151, 620004005, 620004082, 620004083, 620004084, 620006686, 621622602, 621642203, 621734801, 622581501, 622630501, 622634501, 622642401, 622742200, 622742300, 622841001, 622869601, 620000416, 620000417, 620000418

Abbreviations: ICD-10; International Classification of Diseases 10th Revision

© 2024 Ihara Y et al. *JAMA Network Open.*

eTable 3. Characteristics of patients with NSCLC categorized according to therapy type

Patient characteristics	ICI therapy (n = 12,816) No. (%)	Conventional chemotherapy (n = 18,441) No. (%)
Age, mean (SD), y	70.2 (9.1)	70.2 (8.9)
BMI, mean (SD), kg/m^2	21.9 (3.5)	22.1 (3.5)
Sex		
Male	10,287 (80.3%)	14,139 (76.7%)
Female	2,529 (19.7%)	4,302 (23.3%)
Smoking status (current/past) [a]	10,117 (81.7%)	13,793 (77.7%)
Barthel index score (perfect score) [a]	10,893 (88.9%)	16,027 (90.3%)
CCI score		
0–2	4,368 (34.1%)	7,859 (42.6%)
≥ 3	8,448 (65.9%)	10,582 (57.4%)
Histology of NSCLC		
Squamous	2,317 (18.1%)	3,062 (16.6%)
Non-Squamous	10,499 (81.9%)	15,379 (83.4%)
Underlying conditions		
Hypertension	5,809 (45.3%)	7,457 (40.4%)
Diabetes mellitus	2,709 (21.1%)	4,074 (22.1%)
Renal failure	591 (4.6%)	631 (3.4%)
Liver failure	2,198 (17.2%)	2,706 (14.7%)
Cardiovascular disease	4,272 (33.3%)	5,356 (29.0%)
Dyslipidemia	3,176 (24.8%)	4,173 (22.6%)
Medication		
Immunosuppressant	111 (0.9%)	512 (2.8%)
Calendar dates of therapies		
2015/12/01–2019/12/31	4,292 (33.5%)	11,931 (64.7%)
2020/01/01–2023/01/31	8,524 (66.5%)	6,510 (35.3%)

[a] For Smoking status, and Barthel index, there were missing data for 3.6%, and 4.0% of the total patients, respectively.

Abbreviations: NSCLC, non-small cell lung cancer; ICI, Immune checkpoint inhibitor; SD, standard deviation; BMI, body mass index; CCI, Charlson comorbidity index

© 2024 Ihara Y et al. *JAMA Network Open.*

eTable 4. Characteristics of patients with NSCLC categorized according to age

	Age ≤ 64 years		Age 65–79 years		Age ≥ 80 years	
	ICI therapy (n = 2,835)	Conventional chemotherapy (n = 3,938)	ICI therapy (n = 8,290)	Conventional chemotherapy (n = 12,190)	ICI therapy (n = 1,691)	Conventional chemotherapy (n = 2,313)
Patient characteristics	No. (%)	No. (%)	No. (%)	No. (%)	No. (%)	No. (%)
Age, mean (SD), y	56.9 (6.4)	57.1 (6.7)	72.2 (3.9)	72.1 (4.0)	82.9 (2.6)	82.5 (2.4)
BMI, mean (SD), kg/m^2	22.1 (3.7)	22.3 (3.8)	21.9 (3.5)	22.1 (3.4)	22.1 (3.3)	22.2 (3.2)
Sex						
Male	2,239 (79.0%)	2,874 (73.0%)	6,733 (81.2%)	9,492 (77.9%)	1,315 (77.8%)	1,773 (76.7%)
Female	596 (21.0%)	1,064 (27.0%)	1,557 (18.8%)	2,698 (22.1%)	376 (22.2%)	540 (23.3%)
Smoking status (current/past)	2,308 (83.9%)	2,946 (77.7%)	6,610 (82.6%)	9,321 (79.4%)	1,199 (73.9%)	1,526 (69.0%)
Barthel index score (perfect score)	2,501 (91.7%)	3,543 (93.0%)	7,038 (88.8%)	10,587 (90.2%)	1,354 (84.4%)	1,897 (86.5%)
CCI score						
0–2	899 (31.7%)	1,732 (44.0%)	2,864 (34.5%)	5,210 (42.7%)	605 (35.8%)	917 (39.6%)
≥ 3	1,936 (68.3%)	2,206 (56.0%)	5,426 (65.5%)	6,980 (57.3%)	1,086 (64.2%)	1,396 (60.4%)
Histology of NSCLC						
Squamous	311 (11.0%)	462 (11.7%)	1,633 (19.7%)	2,184 (17.9%)	373 (22.1%)	416 (18.0%)
Non-Squamous	2,524 (89.0%)	3,476 (88.3%)	6,657 (80.3%)	10,006 (82.1%)	1,318 (77.9%)	1,897 (82.0%)
Underlying conditions						
Hypertension	835 (29.5%)	1,050 (26.7%)	4,023 (48.5%)	5,190 (42.6%)	951 (56.2%)	1,217 (52.6%)

© 2024 Ihara Y et al. *JAMA Network Open.*

Diabetes mellitus	403 (14.2%)	524 (13.3%)	1,906 (23.0%)	2,955 (24.2%)	400 (23.7%)	595 (25.7%)
Renal failure	67 (2.4%)	58 (1.5%)	389 (4.7%)	442 (3.6%)	135 (8.0%)	131 (5.7%)
Liver failure	475 (16.8%)	521 (13.2%)	1,423 (17.2%)	1,823 (15.0%)	300 (17.7%)	362 (15.7%)
Cardiovascular disease	634 (22.4%)	744 (18.9%)	2,891 (34.9%)	3,713 (30.5%)	747 (44.2%)	899 (38.9%)
Dyslipidemia	384 (13.5%)	522 (13.3%)	2,261 (27.3%)	2,969 (24.4%)	531 (31.4%)	682 (29.5%)
Medication						
Immunosuppressant	21 (0.7%)	113 (2.9%)	70 (0.8%)	345 (2.8%)	20 (1.2%)	54 (2.3%)
Calendar dates of therapies						
2015/12/01–2019/12/31	1,030 (36.3%)	2,794 (70.9%)	2,717 (32.8%)	7,834 (64.3%)	545 (32.2%)	1,303 (56.3%)
2020/01/01–2023/01/31	1,805 (63.7%)	1,144 (29.1%)	5,573 (67.2%)	4,356 (35.7%)	1,146 (67.8%)	1,010 (43.7%)

Abbreviations: NSCLC, non-small cell lung cancer; ICI, Immune checkpoint inhibitor; SD, standard deviation; BMI, body mass index; CCI, Charlson comorbidity index

© 2024 Ihara Y et al. *JAMA Network Open.*

eTable 5. Characteristics of patients with NSCLC categorized according to sex

	Male		Female	
	ICI therapy (n = 10,287)	Conventional chemotherapy (n = 14,139)	ICI therapy (n = 2,529)	Conventional chemotherapy (n = 4,302)
Patient characteristics	No. (%)	No. (%)	No. (%)	No. (%)
Age, mean (SD), y	70.3 (8.9)	70.4 (8.6)	70.1 (9.8)	69.5 (9.9)
BMI, mean (SD), kg/m^2	22.0 (3.4)	22.2 (3.4)	21.6 (3.9)	21.9 (4.0)
Smoking status (current/past)	8,912 (89.7%)	12,117 (89.4%)	1,205 (49.2%)	1,676 (40.0%)
Barthel index score (perfect score)	8,826 (89.5%)	12,305 (90.5%)	2,067 (86.5%)	3,722 (89.6%)
CCI score				
0–2	3,445 (33.5%)	5,938 (42.0%)	923 (36.5%)	1,921 (44.7%)
≥ 3	6,842 (66.5%)	8,201 (58.0%)	1,606 (63.5%)	2,381 (55.3%)
Histology of NSCLC				
Squamous	1,993 (19.4%)	2,673 (18.9%)	324 (12.8%)	389 (9.0%)
Non-Squamous	8,294 (80.6%)	11,466 (81.1%)	2,205 (87.2%)	3,913 (91.0%)
Underlying conditions				
Hypertension	4,778 (46.4%)	5,844 (41.3%)	1,031 (40.8%)	1,613 (37.5%)
Diabetes mellitus	2,343 (22.8%)	3,431 (24.3%)	366 (14.5%)	643 (14.9%)
Renal failure	512 (5.0%)	534 (3.8%)	79 (3.1%)	97 (2.3%)
Liver failure	1,810 (17.6%)	2,096 (14.8%)	388 (15.3%)	610 (14.2%)
Cardiovascular disease	3,556 (34.6%)	4,307 (30.5%)	716 (28.3%)	1,049 (24.4%)
Dyslipidemia	2,555 (24.8%)	3,150 (22.3%)	621 (24.6%)	1,023 (23.8%)

© 2024 Ihara Y et al. *JAMA Network Open.*

Medication				
Immunosuppressant	84 (0.8%)	299 (2.1%)	27 (1.1%)	213 (5.0%)
Calendar dates of therapies				
2015/12/01–2019/12/31	3,437 (33.4%)	9,289 (65.7%)	855 (33.8%)	2,642 (61.4%)
2020/01/01–2023/01/31	6,850 (66.6%)	4,850 (34.3%)	1,674 (66.2%)	1,660 (38.6%)

Abbreviations: NSCLC, non-small cell lung cancer; ICI, Immune checkpoint inhibitor; SD, standard deviation; BMI, body mass index; CCI, Charlson comorbidity index

© 2024 Ihara Y et al. *JAMA Network Open.*

eTable 6. Characteristics of patients with NSCLC categorized according to details on therapy types

Patient characteristics	Chemoimmunotherapy combinations (n = 7,847) No. (%)	ICI monotherapy (n = 4,969) No. (%)	Conventional chemotherapy (n = 18,441) No. (%)
Age, mean (SD), y	68.2 (8.7)	73.5 (8.8)	70.2 (8.9)
BMI, mean (SD), kg/m^2	22.1 (3.5)	21.7 (3.5)	22.1 (3.5)
Sex			
Male	6,355 (81.0%)	3,932 (79.1%)	14,139 (76.7%)
Female	1,492 (19.0%)	1,037 (20.9%)	4,302 (23.3%)
Smoking status (current/past)	6,277 (82.6%)	3,840 (80.3%)	13,793 (77.7%)
Barthel index score (perfect score)	6,910 (91.4%)	3,983 (84.8%)	16,027 (90.3%)
CCI score			
0–2	2,654 (33.8%)	1,714 (34.5%)	7,859 (42.6%)
≥ 3	5,193 (66.2%)	3,255 (65.5%)	10,582 (57.4%)
Histology of NSCLC			
Squamous	1,383 (17.6%)	934 (18.8%)	3,062 (16.6%)
Non-Squamous	6,464 (82.4%)	4,035 (81.2%)	15,379 (83.4%)
Underlying conditions			
Hypertension	3,469 (44.2%)	2,340 (47.1%)	7,457 (40.4%)
Diabetes mellitus	1,602 (20.4%)	1,107 (22.3%)	4,074 (22.1%)
Renal failure	252 (3.2%)	339 (6.8%)	631 (3.4%)

© 2024 Ihara Y et al. *JAMA Network Open.*

Liver failure	1,389 (17.7%)	809 (16.3%)	2,706 (14.7%)
Cardiovascular disease	2,458 (31.3%)	1,814 (36.5%)	5,356 (29.0%)
Dyslipidemia	1,846 (23.5%)	1,330 (26.8%)	4,173 (22.6%)
Medication			
Immunosuppressant	63 (0.8%)	48 (1.0%)	512 (2.8%)
Calendar dates of therapies			
2015/12/01–2019/12/31	1,814 (23.1%)	2,478 (49.9%)	11,931 (64.7%)
2020/01/01–2023/01/31	6,033 (76.9%)	2,491 (50.1%)	6,510 (35.3%)

Abbreviations: NSCLC, non-small cell lung cancer; ICI, Immune checkpoint inhibitor; SD, standard deviation; BMI, body mass index; CCI, Charlson comorbidity index

© 2024 Ihara Y et al. *JAMA Network Open.*

eTable 7. Characteristics of NSCLC patients with and without BMI values

	Without BMI values	With BMI values	Without BMI values	With BMI values
	ICI therapy (n = 12,816)	ICI therapy (n = 1,744)	Conventional chemotherapy (n = 18,441)	Conventional chemotherapy (n = 2,952)
Patient characteristics	No. (%)	No. (%)	No. (%)	No. (%)
Age, mean (SD), y	70.2 (9.1)	70.1 (10.0)	70.2 (8.9)	70.0 (9.5)
BMI, mean (SD), kg/m^2	21.9 (3.5)	—	22.1 (3.5)	—
Sex				
Male	10,287 (80.3%)	1,382 (79.2%)	14,139 (76.7%)	1,959 (66.4%)
Female	2,529 (19.7%)	362 (20.8%)	4,302 (23.3%)	993 (33.6%)
Smoking status (current/past)	10,117 (81.7%)	1,108 (77.6%)	13,793 (77.7%)	1,719 (65.5%)
Barthel index (perfect score)	10,893 (88.9%)	489 (76.8%)	16,027 (90.3%)	935 (82.7%)
CCI				
0–2	4,368 (34.1%)	659 (37.8%)	7,859 (42.6%)	1,211 (41.0%)
≥ 3	8,448 (65.9%)	1,085 (62.2%)	10,582 (57.4%)	1,741 (59.0%)
Histology of NSCLC				
Squamous cell carcinoma	2,317 (18.1%)	305 (17.5%)	3,062 (16.6%)	281 (9.5%)
Non-Squamous cell carcinoma	10,499 (81.9%)	1,439 (82.5%)	15,379 (83.4%)	2,671 (90.5%)
Underlying conditions				
Hypertension	5,809 (45.3%)	731 (41.9%)	7,457 (40.4%)	1,232 (41.7%)
Diabetes mellitus	2,709 (21.1%)	291 (16.7%)	4,074 (22.1%)	554 (18.8%)
Renal failure	591 (4.6%)	77 (4.4%)	631 (3.4%)	101 (3.4%)

© 2024 Ihara Y et al. *JAMA Network Open.*

Liver failure	2,198 (17.2%)	260 (14.9%)	2,706 (14.7%)	525 (17.8%)
Cardiovascular disease	4,272 (33.3%)	518 (29.7%)	5,356 (29.0%)	931 (31.5%)
Dyslipidemia	3,176 (24.8%)	372 (21.3%)	4,173 (22.6%)	627 (21.2%)
Medication				
Immunosuppressant	111 (0.9%)	15 (0.9%)	512 (2.8%)	113 (3.8%)
Calendar dates of therapies				
2015/12/01-2019/12/31	4,292 (33.5%)	557 (31.9%)	11,931 (64.7%)	1,641 (55.6%)
2020/01/01-2023/01/31	8,524 (66.5%)	1,187 (68.1%)	6,510 (35.3%)	1,311 (44.4%)

Abbreviations: NSCLC, non-small cell lung cancer; ICI, Immune checkpoint inhibitor; SD, standard deviation; BMI, body mass index; CCI, Charlson comorbidity index

© 2024 Ihara Y et al. *JAMA Network Open.*

eFigure 1. Distribution of BMI

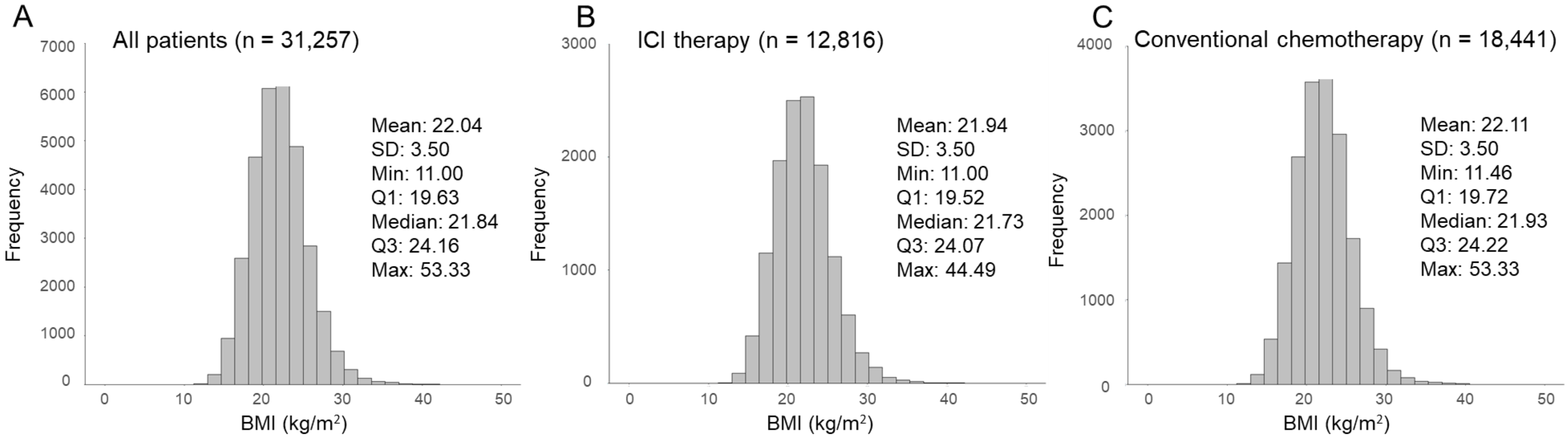

Abbreviations: BMI, body mass index; SD, standard deviation; Q1, first quartile; Q3, third quartile; Min, minimum; Max, maximum

© 2024 Ihara Y et al. *JAMA Network Open.*

eFigure 2. Hazard functions of mortality for patients who received ICI therapy or conventional chemotherapy and difference of hazard of mortality plotted against BMI in sensitivity analyses

Sensitivity analysis 1 on length of period before lung cancer

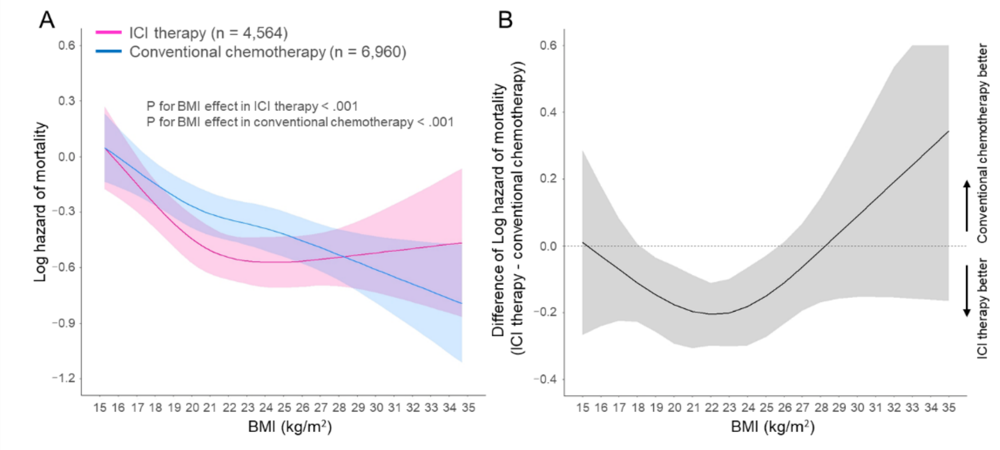

Sensitivity analysis 2 with censoring at time of regimen cross-over

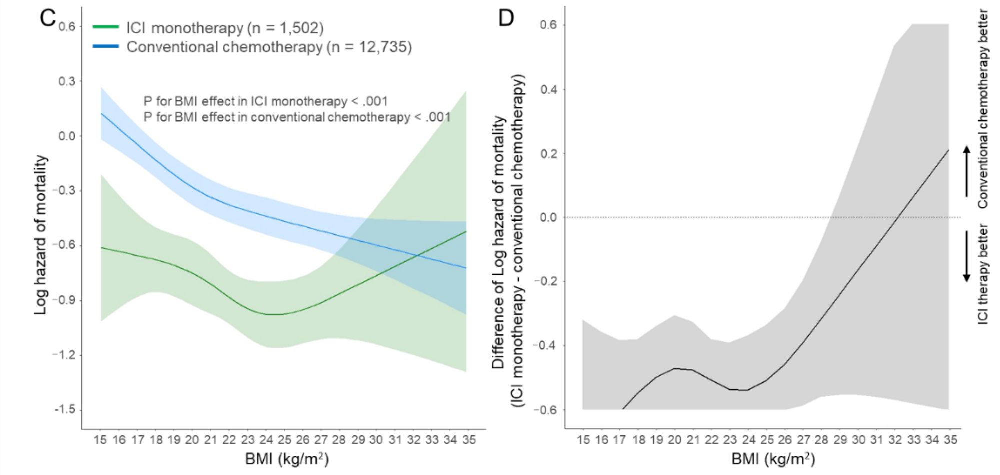

Sensitivity analysis 3 in missing value completion of BMI

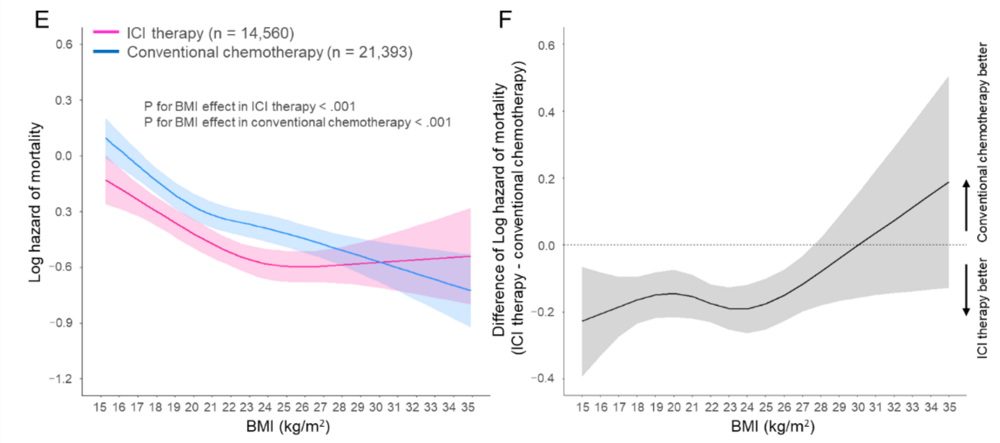

© 2024 Ihara Y et al. *JAMA Network Open.*

eFigure 2 shows the log of the mortality hazard as a function of BMI, stratified by therapeutic type in sensitivity analysis 1, sensitivity analysis 2 and sensitivity analysis 3 populations. A consistent trend was associated with a lower hazard of mortality for patients with a higher BMI in both therapy types for all sensitivity analyses (eFigure 2A, 2C, 2E). However, in sensitivity analysis 2, the mortality hazard was higher for patients with higher BMI. The association of lower mortality in patients treated with ICI compared with those treated with conventional chemotherapy was observed to disappear before and after overweight or obesity in all sensitivity analyses (eFigure2B, 2D, 2F).

Abbreviations: BMI, body mass index; ICI, Immune checkpoint inhibitors

© 2024 Ihara Y et al. *JAMA Network Open.*

eFigure 3. Hazard of mortality as a function of BMI stratified based on details on therapy types

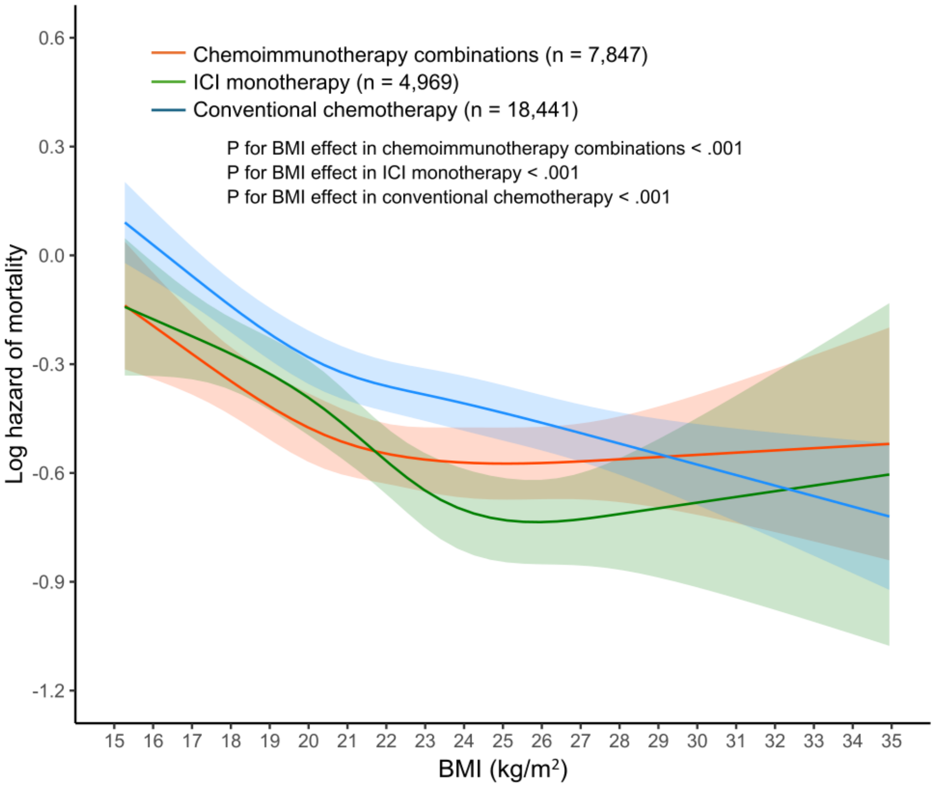

eFigure 3 details the log of the mortality hazard as a function of BMI stratified by therapy type, comparing ICI monotherapy and a combination of ICI and chemotherapy. In patients who are overweight or obese, receiving ICI monotherapy or chemoimmunotherapy combinations was not associated with a lower risk of mortality compared to those receiving conventional chemotherapy.

Abbreviations: BMI, body mass index; ICI, Immune checkpoint inhibitors

© 2024 Ihara Y et al. *JAMA Network Open.*
