# Supplementary material for: Immunotherapy and Overall Survival Among Patients With Advanced Non–Small Cell Lung Cancer and Obesity
Source: JAMA Netw Open. 2024 Aug 2;7(8):e2425363. doi: 10.1001/jamanetworkopen.2024.25363 (PMC11297387; doi:10.1001/jamanetworkopen.2024.25363)
Supplement: Supplement 2. — Data Sharing Statement [file jamanetwopen-e2425363-s002.pdf]

Data Sharing Statement

Ihara. Immunotherapy and Overall Survival Among Patients With Advanced Non–Small Cell Lung Cancer and Obesity. *JAMA Netw Open*. Published August 02, 2024. doi:10.1001/jamanetworkopen.2024.25363

Data
Data available: No

Additional Information
Explanation for why data not available: The database provided by Medical Data Vision Co., Ltd. was used under a license agreement and is not publicly available.
